# Assessing Residential Exposure Risk from Spills of Flowback Water from Marcellus Shale Hydraulic Fracturing Activity

**DOI:** 10.3390/ijerph15040727

**Published:** 2018-04-11

**Authors:** Noura Abualfaraj, Patrick L. Gurian, Mira S. Olson

**Affiliations:** CAEE Department, College of Engineering, Drexel University, 3141 Chestnut St., Philadelphia, PA 19104, USA; plg28@drexel.edu (P.L.G.); mso28@drexel.edu (M.S.O.)

**Keywords:** shale gas, hydraulic fracturing, flowback, risk assessment, drinking water

## Abstract

Identifying sources of concern and risk from shale gas development, particularly from the hydraulic fracturing process, is an important step in better understanding sources of uncertainty within the industry. In this study, a risk assessment of residential exposure pathways to contaminated drinking water is carried out. In this model, it is assumed that a drinking water source is contaminated by a spill of flowback water; probability distributions of spill size and constituent concentrations are fit to historical datasets and Monte Carlo simulation was used to calculate a distribution of risk values for two scenarios: (1) use of a contaminated reservoir for residential drinking water supply and (2) swimming in a contaminated pond. The swimming scenario did not produce risks of concern from a single exposure of 1 h duration, but 11 such 1-h exposures did produce risks of 10^−6^ due to radionuclide exposure. The drinking water scenario over a 30-year exposure duration produced cancer risk values exceeding 10^−6^ for arsenic, benzene, benzo(a)pyrene, heptachlor, heptachlor epoxide, pentachlorophenol, and vinyl chloride. However, this extended exposure duration is probably not realistic for exposure by a spill event. Radionuclides produced risks in the residential drinking water scenario of 10^−6^ in just 8 h, a much more realistic timeline for continual exposure due to a spill event. In general, for contaminants for which inhalation exposure was applicable, this pathway produced the highest risks with exposure from ingestion posing the next greatest risk to human health followed by dermal absorption (or body emersion for radionuclides). Considering non-carcinogenic effects, only barium and thallium exceed target limits, where the ingestion pathway seems to be of greater concern than dermal exposure. Exposure to radionuclides in flowback water, particularly through the inhalation route, poses a greater threat to human health than other contaminants examined in this assessment and should be the focus of risk assessment and risk mitigation efforts.

## 1. Introduction

The increase in the development and production of natural gas over the past two decades in the United States has been an ongoing source of environmental concern [1,2]. Technological advances in drilling techniques have made natural gas extraction from tight, impervious shale formations economically feasible. Shale gas extraction (typically referred to as unconventional drilling) combines directional drilling, which steers the drill bit horizontally along the shale formation allowing each well to access a larger area of rock, and hydraulic fracturing, the process of injecting pressurized water mixed with a proppant (typically sand) and chemical additives in order to create fractures within the rock through which gas can escape. The additives used in hydraulic fracturing fluid make up roughly 0.5–2% of the fluid and act as reducers and surfactants, while the proppant (10–20% of the fracturing fluid) acts to prop open the fractures created in the shale allowing gas to flow to the well [3,4,5,6,7,8].

Sources of environmental and human health concerns from the shale gas industry include: stress on fresh water resources due to the large amount of water (2–5 million gallons) required for each fracturing process [3,4,7,9,10], air quality effects from the release of gas, volatile organic compounds (VOCs), and particulate matter into the atmosphere [1,11,12,13,14,15,16], and water quality effects from surface spills or failures in the subsurface [5,9,17,18,19,20,21,22,23,24,25,26]. Shale gas development is of particular concern in the state of Pennsylvania, as it is currently the US’ second producer of natural gas following Texas, and because the Marcellus shale, which underlies two-thirds of the state, is one of the largest natural gas reservoirs in the country [22,27,28].

Research on the potential mechanisms for drinking water contamination from unconventional drilling, such as accidental spills, inadequate treatment, and improper disposal of waste water, has produced varied results. Some studies [19,21,24] have found evidence of higher levels of thermogenic methane from shale in drinking water wells in closer proximity to unconventional wells. These studies have been criticized for having small sample sizes and for a lack of baseline data collected prior to drilling. Thermogenic methane geochemically consistent with Middle Devonian gases has been reportedly observed in streams in northern Pennsylvania [29]. One study [30] found no statistically significant relationship between methane concentration in drinking water and proximity to wells when examining pre- and post-drilling data provided by Chesapeake Energy Corporation, while another [31] suggests that the thermogenic gas present in drinking water may have originated from shale layers above the Marcellus shale and is, therefore, not an indicator of pollution caused by hydraulic fracturing.

Certain chemicals in hydraulic fracturing fluid and flowback water have the potential to cause severe adverse health effects after chronic or even acute exposure [1,32]. Studies have found that hydraulic fracturing wastewater generally has very high concentrations of salts and total dissolved solids, as well as levels of radionuclides, metals, and organic compounds that could be harmful to human health [32,33,34,35,36,37,38].

The US Environmental Protection Agency’s (EPA) hydraulic fracturing risk assessment report released in 2016 concluded that the industry did not pose a systemic threat to drinking water quality, noting that incidents of failure were relatively rare when considering the total number of wells drilled across the United States [39]. On the other hand, studies of violation and compliance rates for natural gas wells in the state of Pennsylvania have found statistically significant higher rates of environmental violations related to cementing and casing failures, spills, and erosion and sediment control for unconventional wells over conventional wells [40,41,42].

An evaluation of a ground water contamination incident in Bradford County, Pennsylvania (PA) attributed to shale gas development found evidence of dissolved hydrocarbons and inorganics that potentially migrated from nearby wells with inadequate casings and annular pressure measurements exceeding allowable limits [43]. BTEX (benzene, toluene, ethylbenzene, and xylenes) measurements exceeded drinking water standards in groundwater near several spill incidents in Weld County, Colorado (CO), indicating a plausible route for groundwater contamination from hydraulic fracturing activity [44]. Elicitations of expert opinions in the oil and gas industry have shown that the exposure pathways of most concern regarding impacts on drinking water quality and human health are from accidental releases of flowback water or hydraulic fracturing fluid [45,46].

While evidence of systemic negative impacts from shale gas extraction is limited, the literature highlights a degree of uncertainty associated with the shale gas industry, providing a reasonable motivation for examining its potential risk to human health. The goal of this study is to conduct a risk assessment for residential exposure of the general public to a set of carcinogenic and non-carcinogenic chemicals of concern to human health found in flowback water through several ingestion, inhalation, and dermal exposure pathway scenarios in order to better understand potential hazards and provide decision makers with tools to better inform safety practices and failure prevention. Given the amount of uncertainty associated with clean-up and remediation timelines regarding accidental or improper spills, this study utilizes long- and short-term exposure scenarios along with flowback water spill rates, volumes, and contaminant concentrations to develop remedial goals based on long-term Superfund exposure guidelines while also determining appropriate short-term exposure timelines to avoid human health impacts from high priority contaminants with adverse health effects.

## 2. Materials and Methods

The assessment presented in this paper is carried out following the EPA’s recommended Superfund risk assessment guidelines for two residential exposure scenarios: (1) exposure through ingestion, inhalation, and dermal exposure to residential tap water contaminated with flowback water and (2) exposure through swimming in a pond contaminated with flowback water. Details and assumptions for each exposure scenario and pathway are described in this section.

### 2.1. Residential Tap Water Exposure Scenario

The exposure scenario examined in this analysis is modeled as a result of flowback water being accidentally spilled or improperly disposed of directly into a fresh water reservoir that is then used as a source of residential drinking water. The reservoir was assumed to have a volume of 44,000,000 L based on values used in Galada et al. [47], which adopted the EPA scenarios for contamination of rural water supplies by land application of biosolids [48]. The reservoir volume is used to dilute concentrations found in flowback water, assuming that it becomes completely mixed as it enters the fresh water source as described by Equation (1).

(1) $$C_{w}=\left(C_{flowback}\times V_{flowback}\right)/V_{pond}$$

where,

*C_w_* = Chemical concentration in residential drinking water (mg/L)
*C_flowback_* = Chemical concentration in flowback water (mg/L)
*V_flowback_* = Volume of flowback water spilled (L)
*V_pond_* = Volume of drinking water reservoir/holding pond (L)

The contaminant concentration in drinking water is used to estimate exposure dose. In addition to the contaminant concentration, it is necessary to define the exposure duration and frequency parameters in order to determine the daily intake of each contaminant. In this assessment, it is assumed that an adult of average weight will be exposed to drinking water from the scenario described by Equation (1). Residential exposure to this contaminated drinking water source is examined through the following pathways:

1. Ingestion of contaminated drinking water: This scenario assumes direct ingestion of unfiltered residential drinking water. An average daily water ingestion rate of 2.5 L/day was used in this calculation [49].
2. Inhalation of VOCs that may volatilize from the water to the air: This exposure pathway applies to chemicals with a Henry’s Law constant greater than 1 × 10^−5^ atm⋅m^3^/mole and a molecular weight less than 200 g/mole, as they are most likely to volatilize from water during use and contaminate the air. Three carcinogens examined in this assessment meet these criteria, benzene, 1,2-dichloroethane, and vinyl chloride, as well as the radionuclides which may volatilize or aerosolize. This scenario assumes a default volatilization rate of 0.0005 × 1000 L/m^3^ based on an equation defining the relationship between a volatile chemical’s concentration in water and its average volatilized concentration in the air [50]. This includes all household uses of water (showering/bathing, dish washing, cooking, etc.), and assumes an average daily air inhalation rate of 15 m^3^/day [49].
3. Dermal exposure to contaminated water: This pathway examines direct skin contact with contaminated water during showering or bathing. This scenario assumes total skin surface area exposure for 43 min every day based on average values recommended by the *Exposure Factors Handbook* [49].

Daily exposure was assumed for each pathway (Table A1 and Table A2). As standards for chronic exposure are more conservative than acute exposure standards, a chronic exposure was assumed [49]. Values, definitions, and data sources for all the variables required to estimate daily intake can be found in Appendix A (Table A1 and Table A2). From there, exposure durations of concern for each exposure pathway are estimated by determining the exposure duration required for the maximum possible total risk to remain below target values (Total Cancer Risk < 10^−6^ for carcinogens, and Hazard Index < 1.0 for non-carcinogens).

### 2.2. Residential Swimming Exposure Scenario

Similarly to the tap-water scenario, this exposure scenario is modeled as a result of flowback water being accidentally spilled or improperly disposed of directly into 44,000,000 L of fresh water. The pond volume is used to dilute concentrations found in flowback water, assuming that it becomes completely mixed as it enters the fresh water source as described by Equation (1). The exposed individual is then assumed to swim in the pond once for 1 h. In this assessment, the following exposure pathways are considered: dermal exposure to contaminated pond water, accidental ingestion of contaminated pond water during swimming, and inhalation of volatiles in the contaminated pond water during swimming. For a single event, acute exposure parameters are assumed [49]. Exposure durations corresponding to target values of risk (cancer risk < 10^−6^ for carcinogens, and hazard index < 1.0 for non-carcinogens) for each exposure pathway in this scenario are also estimated.

### 2.3. Monte Carlo Simulation

This study utilizes datasets on spill volumes and contaminant concentrations for wastewater generated from hydraulic fracturing activities typically categorized as either flowback or production water. Due to gaps in metadata associated with these datasets, it was not possible, in some cases, to distinguish between flowback and production water. For the purposes of this study, both will be referred to as ‘flowback water’.

Flowback water sampling data from Abualfaraj et al. [33] were used in this analysis. This database reported the concentrations of constituents found in flowback water samples collected from 92 wells in the Marcellus shale region between March 2008 and December 2010. This study [33] prioritized parameters in this dataset based on their concentrations relative to drinking water standards, where high priority was given to constituents with concentrations that exceeded drinking water standards. Potential health effects of ingestion, inhalation, or dermal exposure to contaminants found in flowback water are shown in Table 1.

**Figure 1:**
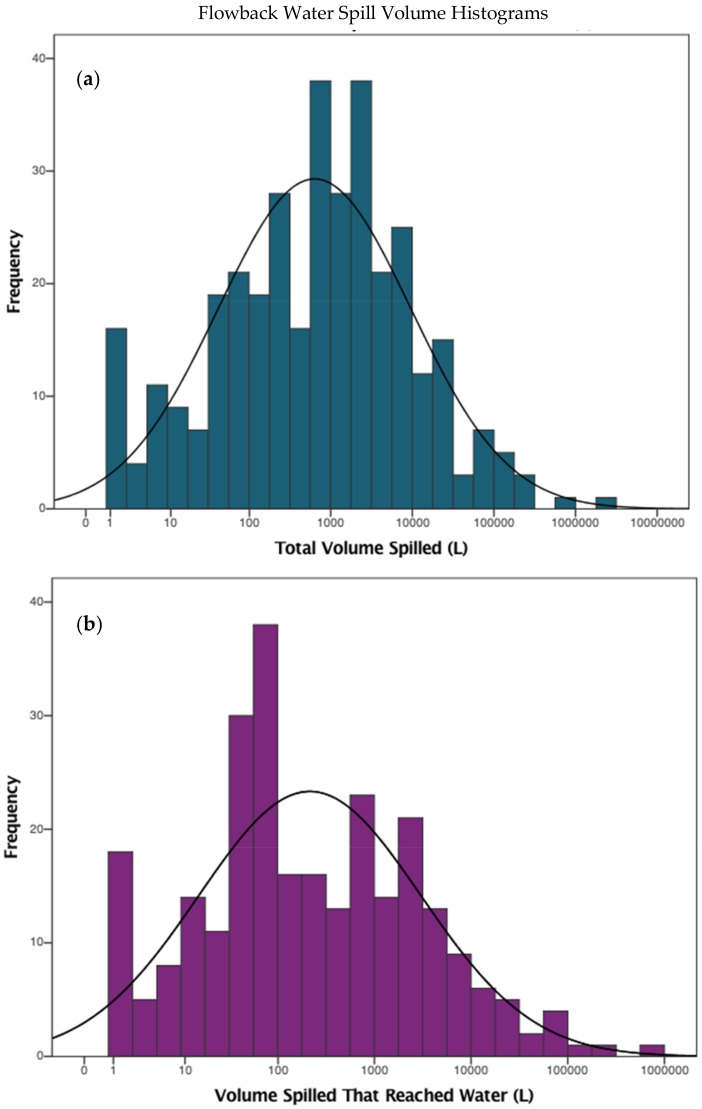
Histogram of flowback water spills that reached water showing (**a**) the total volume spilled and (**b**) the volume that reached water. Annual datasets collected between 2000 and 2016 [54] (Retrieved March 2016).

Flowback water spill volumes were obtained from the United States Coast Guard (USCG) National Response Center’s website, which keeps records of spills and chemical releases each year as well as information about the incident such as the type of contaminant released, the volume spilled, whether the spill reached water, and the portion of the volume that reached water [54]. A distribution of flowback water spill volumes and the volumes that reached water can be found in Figure 1. For this analysis, spill data from 2008 to 2016 were collected and filtered for only spills of flowback water or shale gas production water. As the scenario examined in this assessment involves flowback reaching drinking water, only spills that reached water were considered (Figure 1).

Oracle Crystal Ball (Oracle^®^, Redwood Shores, CA, USA) was used to fit statistical distributions to the spill data and to the flowback water constituent concentration data described above. Crystal Ball was then used to conduct a Monte Carlo analysis using 1000 simulated values for each variable, resulting in a discrete distribution of 1000 results for each cancer risk and hazard quotient calculation. A list of input variables used in the Monte Carlo simulation can be found in Table 2, along with the type of distribution used to fit the data, the number of samples in the distribution, median and 95th percentile values for each distribution, and the data source for each variable.

### 2.4. Residential Risk Assessment for Carcinogens

Equations used for cancer risk following exposure to chemicals in residential drinking water were taken from the EPA’s *Risk Assessment Guidance for Superfund* (RAGS) [50]. This guidance provides methods and equations for conducting human health risk assessments, as well as recommended values for certain parameters required for calculations. The residential drinking water exposure pathways considered are ingestion, inhalation of volatiles, and dermal exposure through showering/bathing. The guidance includes equations for the ingestion and inhalation routes, while specific procedures to assess exposure and risk from the dermal pathway are provided in the *Supplemental Guidance for Dermal Risk Assessment (Part E)* [55]. The *Supplemental Guidance for Inhalation Risk Assessment (Part F)* was also used to calculate toxicity factors for volatile compounds in flowback water (Exhibit B3).

Excess lifetime cancer risk, the increase in the probability of developing cancer over a lifetime, is estimated by multiplying the dose by a chemical-specific toxicity factor (slope factor) for each exposure route. Appendix A presents definitions and inputs for the variables used in cancer risk estimation (Table A1 and Table A2), as well as ingestion cancer toxicity values for the ten carcinogens examined (Table A3). Dermal Absorption Factors and Inhalation Unit Risk values are also presented in Table A3 and are used to calculate dermal and inhalation slope factors (Exhibits B2 and B3). The equations for estimating cancer risk in the EPA’s RAGS guidance for ingestion, dermal, and inhalation risk are shown in Appendix B (Exhibits B1–B3).

The flowback water sampling data utilized in this study include two high-priority radioisotopes (radium-226 and radium-226). Procedures for estimating excess lifetime cancer risk from *Part B* of the *Risk Assessment Guidance for Superfund* are presented in Exhibits B4 [56]. Carcinogenicity slope factors for radionuclides (Table A4) were obtained from the Office of Radiation Programs’ Federal Guidance No. 11 [57]. The *RAGS* guidance does not recommend combining cancer risk from radionuclides with other carcinogens when calculating total risk due to the differences in the equations used and the methods for determining slope factors for radionuclides. Results for radionuclides are, therefore, presented separately.

### 2.5. Residential Toxicity Assessment for Non-Carcinogens

Hazard Quotients, the ratio of exposure to the estimated daily exposure level at which no adverse health effects are likely to occur, are calculated for non-carcinogenic parameters in flowback water. Ingestion, dermal, and inhalation pathways are also considered for non-carcinogens using equations in the EPA’s RAGS [50,55,58]. Definitions and inputs for variables used in this estimation are presented in Appendix A (Table A1 and Table A2). Reference toxicity factors (reference dose) for ingestion and inhalation of contaminants in water are presented in Table A3. Reference doses for dermal exposure are calculated based on the dermal absorption factor and equations presented in Exhibit B2b.

Totals from all three exposure pathways are computed to estimate the total cancer risk and total hazard index for each contaminant. Totals for each pathway for all contaminants are also computed and summed to estimate the total cancer risk and total hazard index (HI), where:

(2) Total cancer risk= Ingestion Risk+Inhalation Risk+Dermal Risk

(3) Total Hazard Index=Ingestion HI+Inhalation HI+Dermal HI

## 3. Results

### 3.1. Residential Exposure from Tap-Water

**Figure 2:**
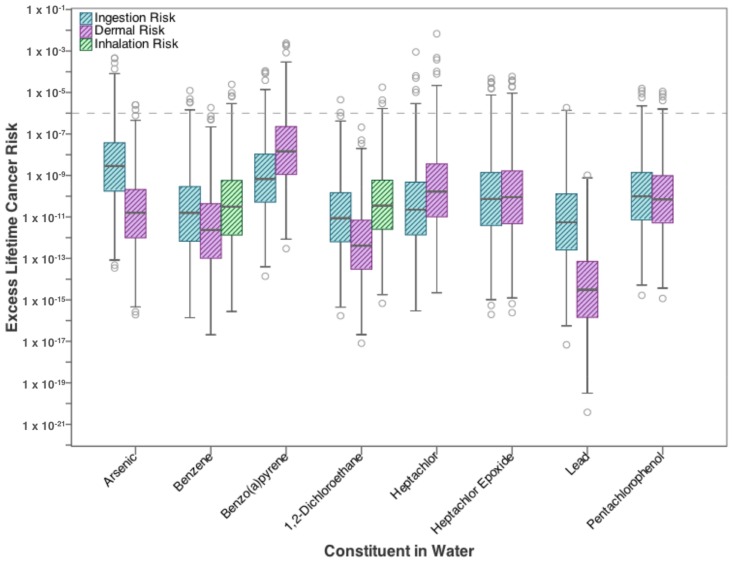
Excess lifetime cancer risk from drinking water exposure to high-priority carcinogenic contaminants found in flowback water.

Excess lifetime cancer risks from ingestion, dermal, and inhalation exposure to the 10 carcinogenic high-priority constituents in flowback water are presented in Figure 2 as a range of values based on the 1000 trials generated by the Monte Carlo simulation. None of the contaminants exceed the target risk of developing cancer (10^−6^) at the median value. However, the upper limit of risk for several constituents exceeds the cancer risk target. Ingestion risk from exposure to arsenic, benzo(a)pyrene, heptachlor, heptachlor epoxide, pentachlorophenol, and vinyl chloride exceed acceptable lifetime cancer risk, with arsenic having the highest 95th percentile value (~10^−4^). This can be expressed as the risk of one incremental increase in cancer occurrence for every ten thousand people exposed to arsenic under similar conditions.

Of the three volatile carcinogens analyzed, only benzene exceeds the target cancer risk, and only between the 95th percentile and the maximum value, where cancer occurrence increases by one for every hundred thousand exposed individuals compared to the unexposed general population. Risk from the dermal exposure route, similarly to the ingestion pathway, exceeds target cancer risk at the upper limit for the following constituents: benzene, benzo(a)pyrene, heptachlor, heptachlor epoxide, and pentachlorophenol.

**Figure 3:**
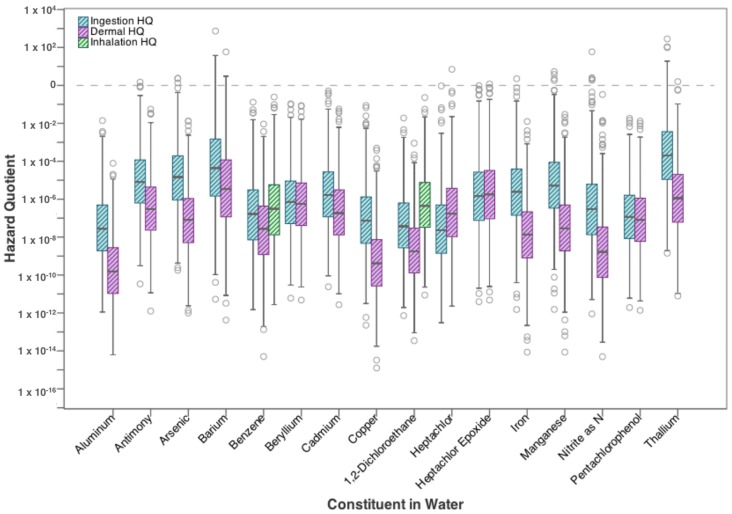
Hazard Quotient from drinking water exposure to high-priority non-carcinogenic contaminants found in flowback water.

Hazard Quotients (HQ) for non-carcinogenic constituents in flowback water are shown in Figure 3. The hazard quotient provides a ratio of the concentration in relation to the reference dose for each parameter with non-carcinogenic adverse health effects, which is then compared to 1, the acceptable target value. It appears from Figure 3 than non-carcinogens found in flowback water pose a much smaller threat to human health than the carcinogenic contaminants in Figure 2. Only two constituents exceeded the target HQ of 1 and only at the upper bound; they are barium and thallium. Risk from both ingestion and dermal exposure to barium exceed the target, while only ingestion of thallium exceeds the target, at the 95th percentile values. Ingestion of barium and thallium can cause nausea and vomiting, liver damage, and nervous system effects (Table 1).

**Figure 4:**
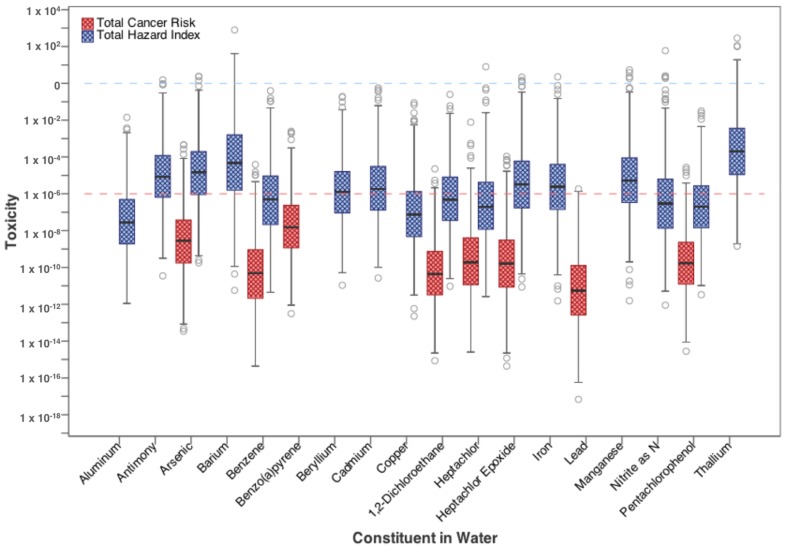
Total cancer risk and total hazard quotient from drinking water ingestion, inhalation, and dermal exposure to high-priority contaminants found in flowback water.

Figure 4 combines risk and HQ values from all three exposure pathways in order to calculate the total cancer risk and total Hazard Index from residential exposure to each contaminant of concern in flowback water. Again, cancer risk and HI values are compared to their target values of 10^−6^ and 1, respectively. The distributions for both cancer risk and HI generally fall below the target limits, with only maximum values and 4th quartile ranges for some parameters exceeding targets. For carcinogens benzene, benzo(a)pyrene, heptachlor, heptachlor epoxide, pentachlorophenol, and vinyl chloride, all have upper-bound concentrations that exceed risks of 10^−6^. For non-carcinogens, similarly to Figure 3, only upper bound (95th percentile) estimates of HI values for barium and thallium exceed 1.

**Figure 5:**
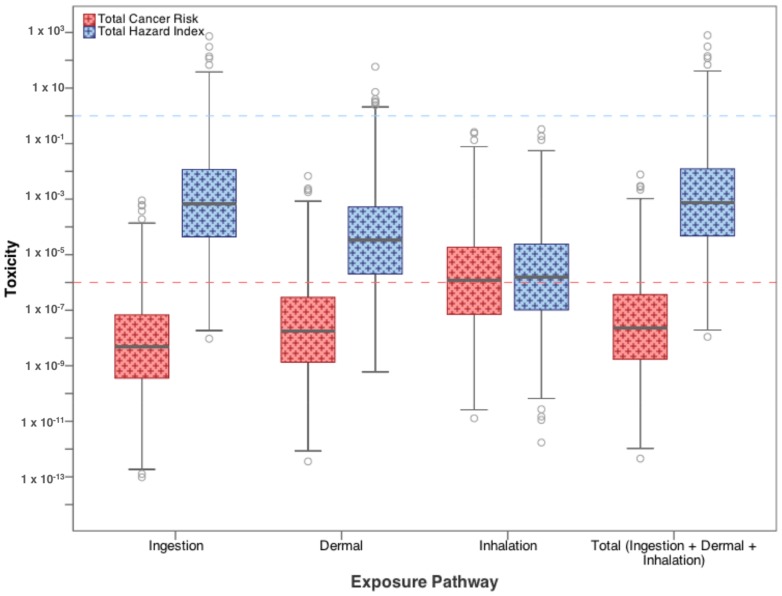
Total cancer risk and hazard index for each drinking water exposure pathway for high-priority contaminants found in flowback water (see Table A3 for list of contaminants included in total).

Figure 5, on the other hand, combines cancer risk and toxicity values of all the carcinogenic and non-carcinogenic constituents for each exposure pathway. In this way, the pathway of most concern can be identified in terms of exposure to high-priority constituents found in flowback water. Figure 5 also includes total cancer risk and the total hazard index for all three pathways combined for all 20 flowback water constituents examined. In both cases, the total risk and total HI are controlled by the exposure pathways of most concern: dermal exposure for carcinogens and ingestion for non-carcinogens.

By varying the exposure duration for each pathway, the maximum possible exposure duration before risk and toxicity values exceed limits were calculated (Table 3 and Table 4). Exposure to carcinogens in flowback water through residential drinking water for as little as 39 days can result in significantly increase cancer risk, assuming the upper bound (95th percentile) on spill volume and contaminant concentration in flowback (Table 3). For non-carcinogens, exposure for up to 31 weeks will not result in adverse health effects with an HI exceeding 1, even at upper bound values for exposure (Table 4).

**Figure 6:**
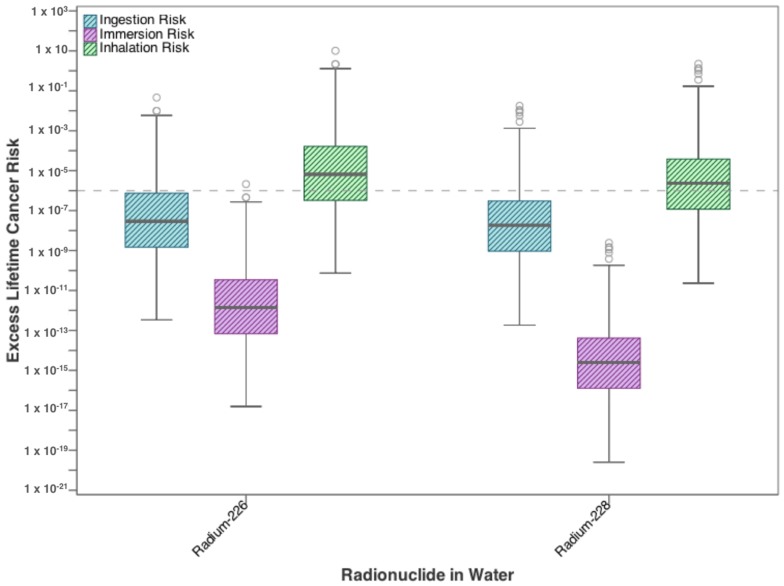
Excess lifetime cancer risk from drinking water exposure to high priority radionuclides found in flowback water.

**Figure 7:**
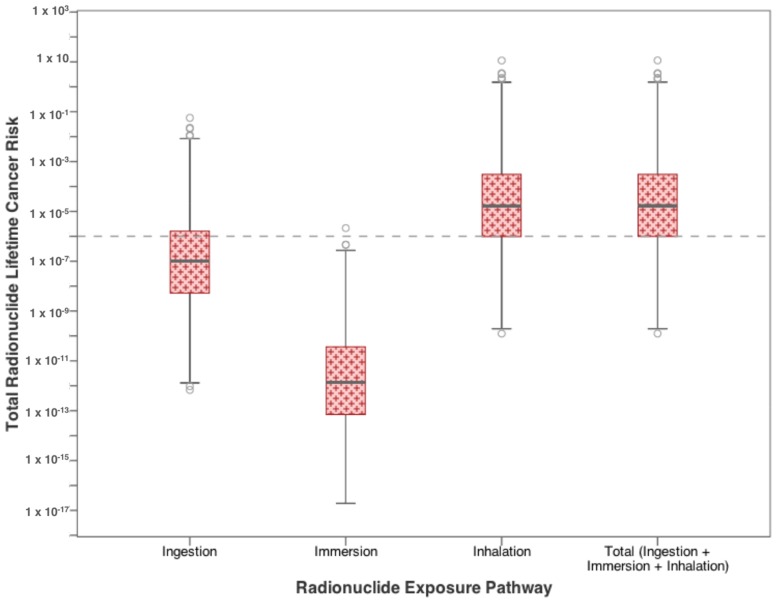
Total cancer risk from drinking water exposure to high-priority radionuclides (radium-226 and radium-228) found in flowback water.

Figure 6 and Figure 7 show the increase in cancer risk from exposure to radionuclides in residential drinking water. In Figure 6, risks from each exposure pathway for radium-226 and radium-228 are presented. For both radioisotopes, inhalation poses the highest risk, where the median values for both parameters exceed the target value of 10^−6^. Median dermal risk from exposure to radionuclides, defined as immersion in water, approached 10^−6^ for radium-226, while radium-228 exceeds the target value at the 75th percentile (Q3). Ingestion risk from both radionuclides exceeds the 10^−6^ target value only at the upper limits (between Q3 and the upper whisker). Combining both radioisotopes shows that the total risk from exposure to radionuclides in water is governed by the inhalation risk (Figure 7). The range for each pathway individually exceeds the target lifetime risk at some level, in fact, the inhalation risk exceeds 10^−6^ even at the 25th percentile value, as does the total risk from all three pathways combined.

### 3.2. Residential Exposure from Swimming

**Figure 8:**
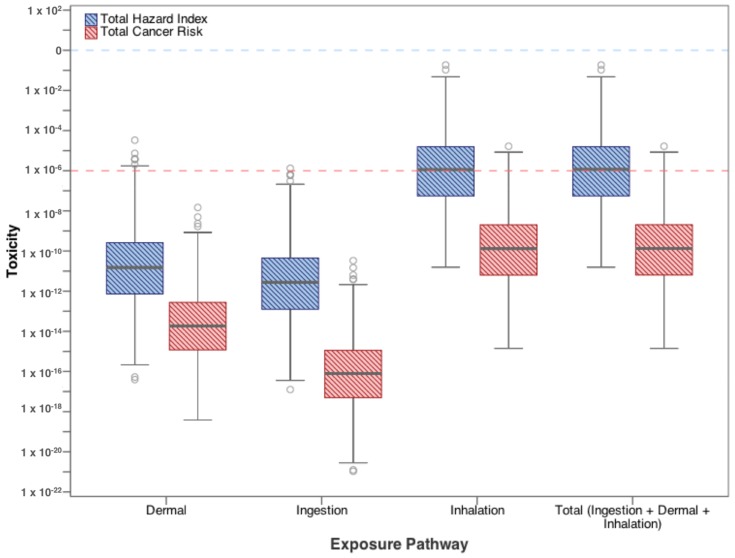
Total cancer risk and hazard index from swimming exposure to high priority contaminants in flowback water.

**Figure 9:**
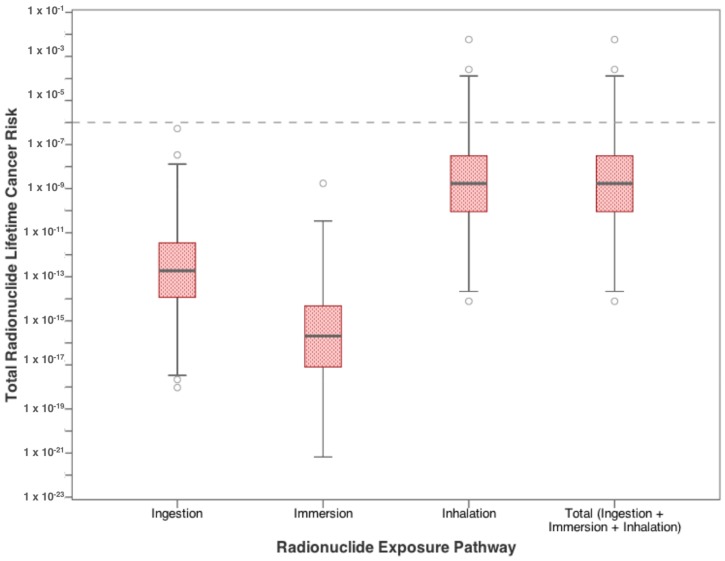
Total cancer risk from exposure pathways to high priority radionuclides from swimming in flowback water.

Only the outlier values for risk from the swimming scenario exceeds 10^−6^ and only from the dermal pathway. In fact, increasing the exposure from 1 h/day for up to 49 days still does not increase the upper bound (95th percentile) risk to above target values (Table 3). Figure 8 shows the total cancer risk and total hazard index from exposure to flowback water in a small fresh water pond by swimming in the pond once for one hour. Figure 9 shows the total cancer risk from exposure to radionuclides in flowback water from swimming in a contaminated pond for one hour. In both figures, the pathway of most concern is the inhalation pathway, which exceeds the target values even at median concentrations, while the dermal and ingestion pathways are below target values.

### 3.3. Uncertainty

In order to better understand the impact each exposure parameter has on the results of the risk model, the Spearman’s Rank Order correlation coefficient was calculated between the input and output variables of the Monte Carlo simulation. The correlation coefficients presented in Table 5 show that the volume of flowback water spilled has the strongest relationship with the total cancer risk and the total hazard index of all the input variables.

## 4. Discussion

The risk assessment conducted in this study provides a priority list for contaminants in flowback water that are likely to pose the greatest threat to human health. In general, the risk of developing cancer from this type of exposure is relatively low, with only values at the upper bound of the Monte Carlo simulation trial results exceeding target limits for excess lifetime cancer risk. Given that the variability in spill volumes was much greater than the variability in concentrations for each chemical, the output of the Monte Carlo simulation is governed by the spill volume. Correlating the input variables with the outcome variables shows that the flowback water volume has the strongest relationship with the total cancer risk and the total hazard index of all the input variables (Table 5). The spill data used in this study included 194 reported spill incidents of flowback that reached water from the National Response Center’s (NRC) data, with volumes ranging from <1 L to 350,000 L. This large range of values results in spill size having the greatest effect on the results of this model. By varying the potential spill volume based on the NRC database, it was possible to calculate a maximum spill volume cutoff value at which no adverse effects from any exposure pathway can be expected. Considering the 30-year drinking water scenario where all other parameters are unchanged, flowback water spills less than 1300 L in volume do not pose a threat to human health from both carcinogenic and non-carcinogenic contaminants. However, for radionuclides, spill volumes as low as 500 L can still result in cancer risk exceeding the target value. This result can be generalized to other scenarios by considering that 500 L represents a 1 in 110,000 dilution in a pond of 44 million L. Scenarios which provide more than this level of dilution before ingestion are likely to contribute minimally to cancer risk even over long periods of time. Other scenarios providing differing dilution amounts or differing exposure duration could be quickly evaluated by noting that a linear cancer risk model is used here and hence risk is proportional to exposure duration and concentration (and so inversely proportional to dilution factor). For the swimming scenario, a spill of 15,000,000 L or greater would be required for total toxicity to exceed target values, however, this is greater than the maximum spill volume reported by the NRC (350,000 L).

Of the ten carcinogens examined in this assessment, benzene, benzo(a)pyrene, heptachlor, heptachlor epoxide, pentachlorophenol, and vinyl chloride are of concern at the upper-bound values. In addition to increasing the risk of developing cancer, chronic exposure to these constituents can have serious adverse effects on human health, including liver and kidney diseases, neurological damage, and compromised immunity. When considering different exposure pathways, dermal exposure through bathing or showering had the highest risk, while inhalation of volatiles from the water does not pose a serious threat considering only three of parameters included in the assessment were considered volatile (benzene, 1,2-dichloroethane, and vinyl chloride).

**Figure 10:**
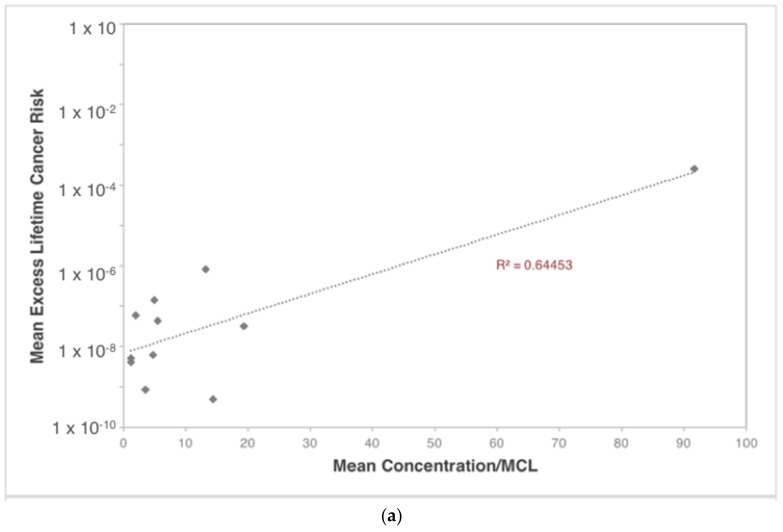

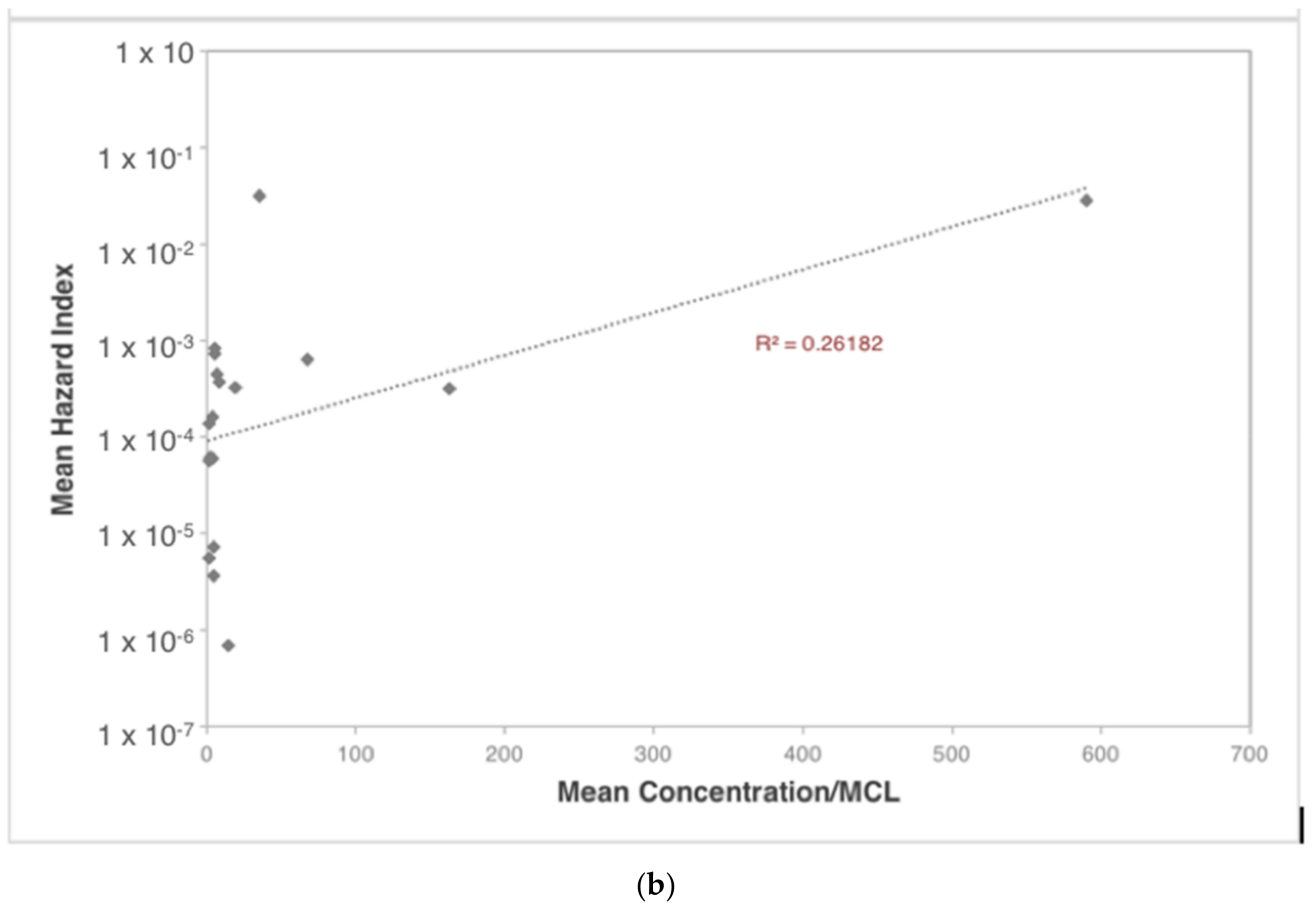
(**a**) Mean excess lifetime cancer risk and (**b**) mean hazard index for high-priority flowback water contaminants in residential drinking water vs. mean concentration/MCL for each contaminant.

It should be noted, however, that while vinyl chloride poses a significant risk at the upper range of the distribution, vinyl chloride concentrations collected from the flowback sampling were mostly not detected as they were either present in flowback water at concentrations below the instrumental detection limit, or were not present at all. Less than 5% of samples had detected values of vinyl chloride, so it is not likely to be a component typically found in flowback water. In addition, the composition of flowback water will vary greatly due to differences in geological, geographical, and biological conditions. The list of high-priority contaminants was developed based on their concentrations relative to drinking water standards; this list will likely vary when applying site-specific conditions or with different samples of flowback water. Figure 10 examines the validity of this prioritization by comparing mean risk and toxicity values for each contaminant to its mean concentration/MCL (Maximum Contamination Level). The data for cancer risk and hazard index generally follow a positive linear relationship between both prioritization methods. The R-squared values, which measure the proportion of the variance predicted by the model, are relatively low for both parameters; (*R*^2^ = 0.64 and 0.26 for cancer risk and hazard index, respectively). While a simple ratio of concentration to MCL is related to risk, this analysis accounts for other factors, such as volatility, dermal absorption, toxicity, etc., that influence risk. Accordingly, prioritization conducted here, based on scenario analyses, is preferred over the concentration/MCL approach proposed in earlier research [33].

Systemic toxicity, expressed as the hazard quotient, from non-carcinogenic contaminants is not generally a concern in the scenarios considered here. Of the parameters considered, barium and thallium exceed the target ratio of 1 at the 95th percentile of the simulated values. Risk from ingestion of these elements poses the greatest threat to human health while toxicity from dermal exposure is much lower (Figure 3, Figure 4 and Figure 5). While not classified as carcinogens, barium and thallium can have adverse health effects following chronic exposure, including gastrointestinal, respiratory, and nervous system effects (Table 1).

Radionuclides, which are known to exist in flowback and produced water as a result of occurring naturally within shale formations, pose a significant risk to human health and increase the likelihood of developing cancer in exposed individuals. Dermal and ingestion risk exceed limits at the higher end of their distributions, while median values for inhalation risk are at unacceptable levels. These exposures are due to radionuclides aerosolizing from water primarily during showering, based on the documented exposure factors developed by the EPA [57]. This exposure poses a significantly greater risk than any other contaminant included in this analysis and, therefore, should not be overlooked.

## 5. Conclusions

Based on the assumed 30-year exposure duration, the scenario presented in this assessment represents an extreme case as this would require a continuous source of contamination with similar volumes and concentrations. Reducing the exposure duration to 5 years reduces all 95th percentile values for total risk and total HI to below target values. The risk of cancer occurrence from radionuclide exposure, however, is still significant (greater than one in one million) for exposure durations as short as 2 weeks. While detailed hydrodynamic modeling is outside the scope of this study, even small water bodies may have residence times well above 2 weeks.

While it may require several years for residential exposure to some carcinogens in flowback water to result in adverse effects, exposure to radionuclides found in natural gas wastewater for only a few hours can pose significant risk to human health. Exposure to contaminated drinking water without notice or taking appropriate steps to remediate or treat the contamination for 30 years may not be a realistic scenario. However, exposure to certain compounds of flowback water for only a few hours or days is a much more likely scenario and can still present adverse effects. While there is evidence of flowback water being illegally disposed of, it is not likely to end up in fresh water supplies without attenuation or degradation, which are not considered in this study. Despite limitations, this assessment provides a preliminary prioritization of risk from different exposure pathways. This provides a useful tool for determining which chemicals will pose the greatest threat to human health, which can better provide insight into necessary precautions and preventative measures for managing and regulating the environmental impacts of shale gas development.

## Figures and Tables

**Table 1:** Health effects from ingestion, dermal, and inhalation exposure to high priority contaminants in flowback water [51,52,53].

Contaminants	Ingestion	Dermal	Inhalation
**Aluminum**	Neurobehavioral alterations; skeletal effects (e.g., osteomalacia)	No known dermal health effects	Impaired lung function and fibrosis
**Antimony**	Nausea, vomiting, diarrhea; stomach cramps	Skin irritation	Irritation to nose, throat, mouth; cough; dizziness
Arsenic *	Gastrointestinal and reproductive effects; possible liver damage	Dermatitis; hyperpigmentation of skin; potential occupational carcinogen	Respiratory distress in animals
**Barium**	Gastroenteritis; muscle spasm; slow pulse	No known dermal health effects	Upper respiratory system effects
Benzene *	Headache, nausea, staggered gait; anorexia, weakness, exhaustion	Skin irritation; dermatitis	Respiratory system effects; dizziness; headache; associated with leukemia
Benzo(a)pyrene *	Causes tumors in animals; birth defects	Dermatitis; regressive verrucae (i.e., warts); skin tumors in animals	Causes tumors in animals
**Beryllium**	Ulcerative gastrointestinal lesions	Dermatitis; skin granulomas	Nasopharyngitis; shortness of breath; labored breathing; chemical pneumonitis
**Cadmium**	Renal tubular damage; increased risk of bone fractures	No known dermal health effects	Decreased lung function; emphysema
**Copper**	Nausea; vomiting; diarrhea	Dermatitis	Irritation to eyes, nose, pharynx; nasal septum perforation
Dibromochloromethane *	Nervous system disorders; liver and kidney disease	Skin irritation; potential occupational carcinogen	Mucous membranes and upper respiratory tract irritation
1,2-Dichloroethane *	Nervous system disorders; liver and kidney disease	Skin lesions; pulmonary tumors; potential occupational carcinogen	Lung effects
Heptachlor *	Liver damage; neurological effects; reproductive system dysfunction	Potential occupational carcinogen	Nervous and immune system effects
Heptachlor Epoxide *	Liver damage; neurological effects; reproductive system dysfunction	Potential occupational carcinogen	Nervous and immune system effects
**Iron**	No known ingestion health effects	No known dermal health effects	Benign pneumoconiosis
Lead *	Malnutrition; constipation, abdominal pain, colic; neurological impairment	No known dermal health effects	Encephalopathy; neurological effects
**Manganese**	Adverse neurological effects	No known dermal health effects	Difficulty breathing; neurological disorder
**Nitrite as N**	Methemoglobinemia; abdominal cramps; vomiting	No known dermal health effects	No known inhalation effects
Pentachlorophenol *	Weakness; nausea; vomiting	Dermatitis; skin lesions; liver effects; renal effects	Irritation to eyes, nose, throat; sneezing, cough; difficulty breathing
**Thallium**	Vomiting; diarrhea; liver and kidney damage	Alopecia (hair loss)	Nervous system effects; pulmonary edema
Vinyl chloride *	Gastrointestinal bleeding; enlarged liver	Skin thickening; frostbite; potential occupational carcinogen	Liver cancer

* Indicates carcinogenic contaminants.

**Table 2:** Distribution median and 95th percentile values for Monte Carlo simulation assumption variables.

Monte Carlo Simulation Input Variables	*N*	Distribution	Median	95th%
Flowback Water Spill Volume (L) ^1^	194	Lognormal	128.00	114,900.00
Aluminum Concentration (mg/L) ^2^	220	Lognormal	0.29	2.80
Antimony Concentration (mg/L) ^2^	186	Triangular	0.05	0.09
Arsenic Concentration (mg/L) ^2^	219	Logistic	0.05	0.09
Barium Concentration (mg/L) ^2^	220	Lognormal	164.00	20,009.00
Benzene Concentration (mg/L) ^2^	123	Lognormal	0.01	0.17
Benzo(a)pyrene Concentration (mg/L) ^2^	111	Logistic	0.01	0.01
Beryllium Concentration (mg/L) ^2^	216	Minimum Extreme	0.02	0.01
Cadmium Concentration (mg/L) ^2^	218	Lognormal	0.01	0.06
Copper Concentration (mg/L) ^2^	219	Lognormal	33,500.00	0.45
1,2-Dichloroethane Concentration (mg/L) ^2^	143	Lognormal	0.01	0.02
Heptachlor Concentration (mg/L) ^2^	73	Pareto	0.01	0.02
Heptachlor Epoxide Concentration (mg/L) ^2^	73	Lognormal	0.01	0.02
Iron Concentration (mg/L) ^2^	233	Lognormal	29.70	178.20
Lead Concentration (mg/L) ^2^	212	Lognormal	0.03	0.20
Manganese Concentration (mg/L) ^2^	220	Lognormal	2.17	12.40
Nitrite as N Concentration (mg/L) ^2^	91	Lognormal	0.11	060.81
Pentachlorophenol Concentration (mg/L) ^2^	111	Weibull	0.01	0.02
Thallium Concentration (mg/L) ^2^	192	Weibull	0.02	0.28
Radium-226 Concentration (PCi/L) ^2^	34	Lognormal	1.30	48,190.20
Radium-228 Concentration (PCi/L) ^2^	30	Lognormal	0.23	4470.00

^1^ Data collected from USCG [54]; ^2^ Data collected from Abualfaraj et al. [33]. Ci = Curie.

**Table 3:** Maximum Exposure Duration (ED) for each scenario and exposure pathway where maximum possible values of cancer risk remain below the target value (<10^−6^).

Exposure Pathway	Maximum Exposure Duration (ED)	
	Drinking Water Scenario	Swimming Scenario
Total Ingestion Cancer Risk	120 days	16.5 years
Total Dermal Cancer Risk	68 days	55 days
Total Inhalation Cancer Risk	1.2 years	2.1 years
Total Risk (Ingestion + Dermal + Inhalation)	39 days	49 days
Total Radionuclide Cancer Risk	8 h	11 days

**Table 4:** Maximum ED for each pathways and scenario where total non-carcinogenic hazard index remains below the target value (<1.0).

Exposure Pathway	Maximum Exposure Duration (ED)	
	Drinking Water Scenario	Swimming Scenario
Ingestion Hazard Quotient	36 weeks	56 years
Dermal Hazard Quotient	17 years	12 years
Inhalation Hazard Quotient	>100 years	>100 years
Hazard Index (Ingestion + Dermal + Inhalation)	31 weeks	9 years

**Table 5:** Spearman’s Rank Order correlation coefficient between input and output variables defined in the Monte Carlo Simulation.

Input Variables	Spearman’s ρ—Output Variables	
	Total Cancer Risk	Total Hazard Index
Flowback Water Spill Volume (L) ^1^	0.975 **	0.915 **
Aluminum Concentration (mg/L)	0.046	0.027
Antimony Concentration (mg/L)	0.005	0.000
Arsenic Concentration (mg/L)	0.016	0.032
Barium Concentration (mg/L)	0.043	0.184 **
Benzene Concentration (mg/L)	0.053 *	0.034
Benzo(a)pyrene Concentration (mg/L)	0.061 *	0.019
Beryllium Concentration (mg/L)	0.028	0.057 *
Cadmium Concentration (mg/L)	0.012	0.039
Copper Concentration (mg/L)	0.007	0.016
Dibromochloromethane Concentration (mg/L)	0.034	0.055 *
1,2-Dichloroethane Concentration (mg/L)	0.048	0.057 *
Heptachlor Concentration (mg/L)	0.025	0.021
Heptachlor Epoxide Concentration (mg/L)	0.008	0.014
Iron Concentration (mg/L)	0.006	0.017
Lead Concentration (mg/L)	0.019	0.018
Manganese Concentration (mg/L)	0.010	0.022
Nitrite as N Concentration (mg/L)	0.013	0.022
Pentachlorophenol Concentration (mg/L)	0.032	0.030
Thallium Concentration (mg/L)	0.023	0.157 **
Vinyl chloride Concentration (mg/L)	0.015	0.016
**Input Variables**	**Total Radionuclide Cancer Risk**	
Flowback Water Spill Volume (L) ^1^	0.901 **	
Radium-226 Concentration (PCi/L)	0.362 **	
Radium-228 Concentration (PCi/L)	0.142 **	

** Correlation significant at the 0.01 level (1-tailed); * Correlation is significant at the 0.05 level (1-tailed); ^1^ Strong correlation (Spearman’s ρ > 0.7). Ci = Curie.

## Appendix A

**Table A1:** Definitions for variables used in cancer risk and hazard quotient calculations.

Variable	Variable Description	Source
*ABS_GI_*	Fraction absorbed in gastrointestinal tract (dimensionless)	[33]
*AT*	Averaging time (70 years)	[55]
*B*	Correlation coefficient (dimensionless)	[55]
*BW*	Average adult body weight (80 kg)	[55]
*DAD*	Dermal absorbed dose (mg/kg⋅day)	[55]
*C_w_*	Chemical concentration in water (mg/cm^3^)	Table 2; Equation (1)
*FA*	Fraction absorbed water (dimensionless)	[59]
*K*	Volatilization rate (0.5 m^3^/L)	[55]
*K_P_*	Dermal permeability coefficient of compound in water (cm/h)	[49]
*IUR*	Inhalation unit risk	Table A3
*RfC_i_*	Inhalation reference concentration (mg/m^3^)	Table A3
*RfD_ABS_*	Absorbed reference dose (mg/kg⋅day)	Table A3; Exhibit B2b
*RfD_i_*	Inhalation reference dose (mg/kg⋅day)	Table A3; Exhibit B3
*RfD_O_*	Oral reference dose (mg/kg⋅day)	Table A3
*SF_ABS_*	Absorbed slope factor (mg/kg⋅day)^−1^	Table A3; Exhibit B2b
*SF_i_*	Inhalation slope factor (mg/kg⋅day)^−1^	Table A3; Exhibit B3
*SF_imm_*	Immersion slope factor (mg/kg⋅day)^−1^	Table A4
*SF_O_*	Oral slope factor (mg/kg⋅day)^−1^	Table A3

**Table A2:** Inputs and definitions for exposure variable assumptions used in cancer risk and hazard quotient calculations.

Variable	Variable Description	Value	Source
*AT*	Averaging time (years)	70 years	[55]
*BW*	Average adult body weight (kg)	80 kg	[55]
*ED*	Exposure duration (years)	30 years	[59]
*EF*	Exposure frequency (days/year)	350 days/year	[49]
*EV*	Event frequency (events/day)	1 event/day	[49]
*IR_w_*	Daily water ingestion rate (L/day)	2.5 L/day	[49]
*IR_a_*	Daily air inhalation rate (m^3^/day)	15 m^3^/day	[49]
*SA*	Exposed skin surface area (cm^2^)	19,652 cm^2^ (avg. adult skin surface area)	[49]
*t*^*^	Time to reach steady-state (h)	Chemical specific	Exhibit B3
*t_event_*	Event duration (h/event)	Typical adult exposure = 0.71 h/day	[49]
*τ_event_*	Lag time per event (h/event)	Chemical specific	Exhibit B3

**Table A3:** Toxicity values for each parameter in water.

Chemical	*SF_o_* (mg/kg-day)^−1^	*RfD_o_* (mg/kg-day)	*ABS_GI_*	*IUR* (μg/m^3^)	*RfC_i_* (mg/m^3^)
Aluminum	-	1.0	1	-	5.0 × 10^−3^
Antimony	-	4.0 × 10^−4^	0.15	-	-
Arsenic	1.5	3.0 × 10^−4^	1	4.3 × 10^−3^	1.5 × 10^−5^
Barium	-	2.0 × 10^−1^	0.07	-	5.0 × 10^−4^
Benzene	5.5 × 10^−2^	4.0 × 10^−3^	1	7.8 × 10^−6^	3.0 × 10^−2^
Benzo(a)pyrene	7.3	-	1	1.10 × 10^−3^	-
Beryllium	-	2.0 × 10^−3^	0.007	2.4 × 10^−3^	2.0 × 10^−5^
Cadmium	-	5.0 × 10^−4^	0.05	1.8 × 10^−3^	1.0 × 10^−5^
Copper	-	4.0 × 10^−2^	1	-	-
Dibromochloromethane	8.4 × 10^−2^	2.0 × 10^−2^	1	-	-
1,2-Dichloroethane	9.1 × 10^−2^	6.0 × 10^−3^	1	-	-
Heptachlor	4.5	5.0 × 10-^4^	1	2.6 × 10^−5^	7.0 × 10^−3^
Heptachlor Epoxide	9.1	1.3 × 10^−5^	1	1.3 × 10^−3^	-
Iron	-	7.0 × 10^−1^	1	2.6 × 10^−3^	-
Lead	8.5 × 10^−3^	-	1	-	-
Manganese	-	2.4 × 10^−2^	1	1.2 × 10^−5^	-
Nitrite as N	-	1.0 × 10^−1^	1	-	5.0 × 10^−5^
Pentachlorophenol	4.0 × 10^−1^	5.0 × 10^−3^	1	-	-
Thallium	-	1.0 × 10^−5^	1	5.1 × 10^−6^	-
Vinyl Chloride	7.2 × 10^−1^	3.0 × 10^−3^	1	-	-

Data Sources: [51,60].

**Table A4:** Toxicity values for radionuclides in water.

Radionuclide	*SF_o_* (Risk/PCi)	*SF_i_* (Risk/PCi)	*SF_imm_* (Risk/year per PCi/L)	Source
Radium-226	3.8 × 10^−10^	2.82 × 10^−8^	6.27 × 10^−14^	[57]
Radium-228	1.04 × 10^−9^	4.37 × 10^−8^	5.02 × 10^−16^	[57]

## Appendix B

*Exhibit B1a. Equations for estimating cancer risk and hazard quotient following ingestion of contaminants in residential drinking water [50]*. $\text{Ingestion Cancer Risk}=\mathrm{CDI}\times {\mathrm{SF}}_{o}$ Where: $\text{Chronic Daily Intake}\;\left(\mathrm{CDI}\right)=\frac{C_{w}\times IR_{w}\times EF\times ED}{AT\times 365\left(\mathrm{days}/\mathrm{year}\right)\times BW}$ $\text{Ingestion Hazard Quotient}=\;\mathrm{CDI}/{\mathrm{RfD}}_{o}$ Where: $\mathrm{Chronic}\;\mathrm{Daily}\;\mathrm{Intake}\;\left(\mathrm{CDI}\right)=\frac{C_{w}\times IR_{w}\times EF\times ED}{ED\times 365\left(\mathrm{days}/\mathrm{year}\right)\times BW}$

*Exhibit B1b. Sample calculation estimating cancer risk and hazard quotient following ingestion of residential drinking water with median concentration of Benzene [50]*.

$$Median\;concentration\;of\;benzene\;in\;drinking\;water=3.75\times 10^{-7}\;mg/L$$

For carcinogenic effect: $\mathrm{Chronic}\;\mathrm{Daily}\;\mathrm{Intake}\;\left(\mathrm{CDI}\right)=\frac{3.75\times 10^{-7}\times 2.5\times 350\times 30}{70\times 365\times 80}=4.8\times 10^{-9}$ $\text{Ingestion Cancer Risk}=\mathrm{CDI}\times {\mathrm{SF}}_{o}=4.8\times 10^{-9}\times 5.5\times 10^{-2}=2.65\times 10^{-10}$ For non-carcinogenic effect: $\mathrm{Chronic}\;\mathrm{Daily}\;\mathrm{Intake}\;\left(\mathrm{CDI}\right)=\frac{3.75\times 10^{-7}\times 2.5\times 350\times 30}{30\times 365\times 80}=1.12\times 10^{-8}$ $\text{Ingestion Hazard Quotient}=\;CDI/RfD_{o}=\;1.12\times 10^{-8}/4.0\times 10^{-3}=2.8\times 10^{-6}$

*Exhibit B2a. Equations for estimating cancer risk and hazard quotient following dermal exposure to contaminants in residential drinking water [55]*. $Dermal\;Cancer\;Risk=\;DAD\;\times \;SF_{ABS}$ Where: $DAD_{for\;inorganics}=\;\frac{C_{w}\times K_{p}\times t_{event}\times EV\times ED\times EF\times SA}{AT\times 365\left(\mathrm{days}/\mathrm{year}\right)\times BW}$ If $t_{event}\leq t^{*}$, then: $DAD_{for\;organics}=\frac{2\times FA\times K_{p}\times C_{w}\times \sqrt{\frac{6\times \tau_{event}\times t_{event}}{\pi }}\times EV\times ED\times EF\times SA}{AT\times 365(days/year)\times BW}$ If $t_{event}>t^{*}$, then: $DAD_{for\;organics}=$ $\frac{FA\times K_{p}\times C_{w}\times \left[\frac{t_{event}}{\left(1+B\right)}+\left(2\tau_{event}\times \left(\frac{1+3B+3B^{2}}{{\left(1+B\right)}^{2}}\right)\right)\right]\times EV\times ED\times EF\times SA}{AT\times 365(days/year)\times BW}$ $Dermal\;Hazard\;Quotient=DAD/RfD_{ABS}$ Where: $DAD_{for\;inorganics}=\frac{C_{w}\times K_{p}\times t_{event}\times EV\times ED\times EF\times SA}{ED\times 365(days/year)\times BW}$ If $t_{event}\leq t^{*}$, then: $DAD_{for\;organics}=\frac{2\times FA\times K_{p}\times C_{w}\times \sqrt{\frac{6\times \tau_{event}\times t_{event}}{\pi }}\times EV\times ED\times EF\times SA}{ED\times 365(days/year)\times BW}$ If $t_{event}>t^{*}$, then: $DAD_{for\;organics}=$ $\frac{FA\times K_{p}\times C_{w}\times \left[\frac{t_{event}}{\left(1+B\right)}+\left(2\tau_{event}\times \left(\frac{1+3B+3B^{2}}{{\left(1+B\right)}^{2}}\right)\right)\right]\times EV\times ED\times EF\times SA}{ED\times 365(days/year)\times BW}$

*Exhibit B2b. Additional equations for estimating cancer risk and hazard quotient following dermal exposure to contaminants in residential drinking water [55]*. Toxicity factors for dermal absorption: $SF_{ABS}=SF_{o}/ABS_{GI}$        [55] $RfD_{ABS}=RfD_{o}\times ABS_{GI}$        [55] Lag time per event τ_event_ (h/event): $\tau_{event}=0.105\times 10^{\left(0.0056\times MW\right)}$ Where: MW=Molecular Weight (g/mole)    (Chemical Specific) Time to reach steady state t^*^ (h): If B≤0.6, then: $t^{*}=2.4\tau_{event}$ If B>0.6, then: $t^{*}=\left(b-\sqrt{b^{2}-c^{2}}\right)\frac{I_{sc}{}^{2}}{I_{sc}\times 10^{\left(-2.8-\left(0.0056\times MW\right)\right)}}$ Where: B=Ratio of the permeability coefficient of a compound through the stratum corneum relative to its permeability coefficient across the viable epidermis (dimensionless) b, c=Correlation coefficients $B=K_{p}\sqrt{MW/2.6}$ $b=\frac{2{\left(2+B\right)}^{2}}{\pi }-c$ $c=\frac{1+3B+3B^{2}}{3\left(1+B\right)}$

*Exhibit B2c. Sample calculation estimating cancer risk and hazard quotient following dermal exposure to flowback water with median concentration of Benzene (full hand exposure for 3 h) [55]*.

$$Median\;concentration\;of\;benzene\;in\;flowback\;water=\;2.5\times 10^{-3}\;mg/L$$

$$SF_{ABS}=SF_{o}/ABS_{GI}=5.5\times 10^{-2}/1=5.5\times 10^{-2}$$

$$RfD_{ABS}=RfD_{o}\times ABS_{GI}=4.0\times 10^{-3}\times \;1\;=\;4.0\times 10^{-3}$$

Lag time per event *τ_event_ (h/event)*:

$$\tau_{event}=0.105\times 10^{\left(0.0056\times MW\right)}=2.87\times 10^{-1}$$

Time to reach steady state *t^*^ (h)*: If B≤0.6, then:

$$t^{*}=2.4\tau_{event}$$

$B=Dimensionless\;ratio\;of\;permeability=K_{p}\times \sqrt{MW/2.6}=5.06\times 10^{-2}<0.6$

$$t^{*}=2.4\tau_{event}=2.4\times 2.87\times 10^{-1}=6.9\times 10^{-1}$$

$$t_{event}=3\;h>t^{*}$$

For carcinogens: If $t_{event}>t^{*}$, then:

$$DAD_{for\;organics}=\frac{FA\times K_{p}\times C_{w}\times \left[\frac{t_{event}}{\left(1+B\right)}+\left(2\tau_{event}\times \left(\frac{1+3B+3B^{2}}{{\left(1+B\right)}^{2}}\right)\right)\right]\times EV\times ED\times EF\times SA}{AT\times 365(days/year)\times BW}$$

$$\frac{1\times 0.0149\times 0.0025\left[\frac{3}{\left(1+0.0506\right)}+\left(2\times 0.287\times \left(\frac{1+3\left(0.0506\right)+3{\left(0.0506\right)}^{2}}{{\left(1+\left(0.0506\right)\right)}^{2}}\right)\right)\right]\times 1\times 4\times 52\times 491}{70\times 365(days/year)\times 80}=6.44\times 10^{-9}$$

$$Dermal\;Cancer\;Risk=\;DAD\;\times \;SF_{ABS}=6.44\times 10^{-9}\times \;5.5\times 10^{-2}=3.5\times 10^{-10}$$

For non-carcinogens: If $t_{event}>t^{*}$, then:

$$DAD_{for\;organics}=\frac{FA\times K_{p}\times C_{w}\times \left[\frac{t_{event}}{\left(1+B\right)}+\left(2\tau_{event}\times \left(\frac{1+3B+3B^{2}}{{\left(1+B\right)}^{2}}\right)\right)\right]\times EV\times ED\times EF\times SA}{ED\times 365(days/year)\times BW}$$

$$=\frac{1\times 0.0149\times 0.0025\left[\frac{3}{\left(1+0.0506\right)}+\left(2\times 0.287\times \left(\frac{1+3\left(0.0506\right)+3{\left(0.0506\right)}^{2}}{{\left(1+\left(0.0506\right)\right)}^{2}}\right)\right)\right]\times 1\times 4\times 52\times 491}{4\times 365(days/year)\times 80}=1.13\times 10^{-7}$$

$$Dermal\;Hazard\;Quotient=DAD/RfD_{ABS}=1.13\times 10^{-7}/4.0\times 10^{-3}=2.8\times 10^{-5}$$

*Exhibit B3a. Equations for estimating cancer risk and hazard quotient following inhalation of volatile contaminants in residential drinking water [50,58]*. $Inhalation\;Cancer\;Risk=CDI\times SF_{i}$ Where: $\mathrm{Chronic}\;\mathrm{Daily}\;\mathrm{Intake}\;\left(\mathrm{CDI}\right)=\frac{C_{w}\times IR_{a}\times EF\times ED}{AT\times 365\left(\mathrm{days}/\mathrm{year}\right)\times BW}$ $Inhalation\;Hazard\;Quotient=\;CDI/RfD_{i}$ Where: $\mathrm{Chronic}\;\mathrm{Daily}\;\mathrm{Intake}\;\left(\mathrm{CDI}\right)=\frac{C_{w}\times IR_{a}\times EF\times ED}{ED\times 365\left(\mathrm{days}/\mathrm{year}\right)\times BW}$ Toxicity factors for dermal absorption: $SF_{i}=IUR\times 70/15\times 0.001$         [61] $RfD_{i}=RfC_{i}\times 15/70$             [62]

*Exhibit B3b. Sample calculation estimating cancer risk and hazard quotient following inhalation of median concentration of Benzene from residential drinking water [50,58]*.

$$Median\;concentration\;of\;benzene\;in\;drinking\;water=3.75\times 10^{-7}\;mg/L$$

For carcinogenic effect: $\mathrm{Chronic}\;\mathrm{Daily}\;\mathrm{Intake}\;\left(\mathrm{CDI}\right)=\frac{3.75\times 10^{-7}\times 15\times 350\times 30}{70\times 365\times 80}=2.8\times 10^{-8}\;$ $Inhalation\;Cancer\;Risk=CDI\times SF_{i}=2.8\times 10^{-8}\;\times \;3.64\times 10^{-2}=1.05\times 10^{-9}$ For non-carcinogenic effect: $\mathrm{Chronic}\;\mathrm{Daily}\;\mathrm{Intake}\;\left(\mathrm{CDI}\right)=\frac{3.75\times 10^{-7}\times 15\times 350\times 30}{30\times 365\times 80}=6.7\times 10^{-8}$ $Inhalation\;Hazard\;Quotient=\;CDI/RfD_{i}=\;6.7\times 10^{-8}/6.43\times 10^{-3}=1.05\times 10^{-5}$

*Exhibit B4a. Equations for estimating cancer risk from exposure to radionuclides in water [62,63,64,65,66]*. $Radionuclide\;Ingestion\;Cancer\;Risk=C_{w}\times EF\times ED\times IR_{w}\times SF_{o}$ $Radionuclide\;Immersion\;Cancer\;Risk=C_{w}\times EF\times ED\times IR_{w}\times SF_{imm}$ $Radionuclide\;Inhalation\;Cancer\;Risk=C_{w}\times EF\times ED\times IR_{a}\times SF_{i}$

*Exhibit B4b. Sample calculation estimating cancer risk from exposure to median concentration of Radium-226 in drinking water [50,56]*.

$$Median\;concentration\;of\;radium-226\;in\;drinking\;water=1.94\times 10^{-2}\;PCi/L$$

Radionuclide Ingestion Cancer Risk $=\;1.94\times 10^{-2}\times \;350\;\times \;30\;\times \;2.5\;\times \;3.85\times 10^{-10}=1.96\times 10^{-7}$ Radionuclide Immersion Cancer Risk $=1.94\times 10^{-2}\;\times \;350\;\times \;30\;\times \;2.5\;\times \;6.27\times 10^{-14}=3.19\times 10^{-11}$ Radionuclide Inhalation Cancer Risk
$=1.94\times 10^{-2}\;\times \;350\;\times \;30\;\times \;15\;\times \;2.82\times 10^{-8}=8.62\times 10^{-5}$
